# Live and carcass production traits for progeny of purebred sires in comparison with the clone of a USDA prime yield grade one carcass

**DOI:** 10.1093/tas/txad041

**Published:** 2023-04-24

**Authors:** Jessica L Sperber, David G Lust, Gregg O Veneklasen, Dean E Hawkins, Trent J McEvers, Ty E Lawrence

**Affiliations:** Department of Agricultural Sciences, West Texas A&M University, Canyon, TX 79016, USA; Department of Agricultural Sciences, West Texas A&M University, Canyon, TX 79016, USA; Timber Creek Veterinary Hospital, Canyon, TX 79015, USA; Department of Agricultural Sciences, West Texas A&M University, Canyon, TX 79016, USA; Department of Agricultural Sciences, West Texas A&M University, Canyon, TX 79016, USA; Department of Agricultural Sciences, West Texas A&M University, Canyon, TX 79016, USA

**Keywords:** carcass, cattle, clone, terminal sire test

## Abstract

Cloning is a technology by which an animal’s tissue can be salvaged and replicated. Carcasses that grade USDA prime–yield grade 1 (P1) represent a rare and antagonistic outcome and are a goal for terminal sire selection in the United States. A terminal sire progeny test generated offspring for a crossbred bull (14% Zebu, 86% Angus; ALPHA), born in 2012 via somatic cell nuclear transfer (SCNT) from a carcass that graded P1. ALPHA progeny (steers and heifers) were compared against progeny of three purebred (Angus; Charolais; Simmental) reference sires. Live production traits included weaning weight, morbidity, mortality, and days on feed; carcass traits included abscessed liver frequency and lung lesion frequency, individual quality and yield grade (YG) parameters, and carcass value. Observed carcass traits for progeny from the Angus, Charolais, and Simmental sires were reflective of the carcass outcomes expected for each sire’s respective breed. Calves sired by the Angus were the earliest maturing indicated by the youngest chronological age at harvest (*P* ≤ 0.02) concomitant with the most backfat (*P <* 0.01), and the greatest marbling scores (*P* < 0.01). Calves sired by the Charolais had the heaviest carcass weight (*P* = 0.04), greatest cutability as assessed by USDA calculated YG (*P *< 0.01) and were the heaviest muscled based on “longissimus” muscle area (*P *< 0.01). ALPHA-sired calves were the most similar in carcass outcomes to calves sired by the Simmental, combining advantageous quality and yield parameters to produce an intermediate for carcass quality and yield. The economic value of moderate carcass outcomes is reflected in the carcass value per century weight, in which ALPHA-sired steers tended (*P* = 0.07) to be of the greatest value compared to other sire groups. ALPHA progeny performed comparably to high-performing reference sires for terminal sire production traits and the P1 genetics in which ALPHA was cloned have economical and biological value in modern U.S. beef production.

## INTRODUCTION

Beef producers continuously search for production opportunities that will improve the economic value of their herd. Herd improvement can result from both environmental and genetic influences. One genetic technology is somatic cell nuclear transfer (SCNT), commonly termed “cloning”. The cloned individual will be an identical genetic copy of the animal from which the DNA was derived (Vajta and Gjerris, 2006). Having the ability to salvage and replicate genetic material is particularly beneficial for genetic outcomes that are rarely found within populations but that are highly desired by the producer. The combination of a USDA prime quality grade (QG) and a USDA yield grade 1 (P1) represents a rare and antagonistic outcome, observed in only 0.07% of the U.S. beef population (Boykin et al., 2017) that combines two premium carcass outcomes (AMS, 2018) of economic value for cattle producers.

Sire selection is a strategy for genetic selection, with the annual parentage per sire typically ranging from 20 to 25 calves through natural breeding, and increasing with implementation of reproductive technologies (Hammack, 2007). A terminal sire system is a breeding program in which no replacement heifers are retained for breeding purposes (Gregory and Cundiff, 1980). Specific live and carcass production traits are associated with breed type. Historically, the Angus breed was associated with early maturing physiology and an easy fleshing ability, resulting in carcasses with increased external fat thickness and superior intramuscular fat (Marshall, 1994). The Charolais breed has a high potential for growth resulting in large hot carcass weights (HCWs), increased cutability, reduced overall external fat depth, and reduced marbling (Marshall, 1994; Hammack, 2007). Historically, the Simmental breed was associated with increased cutability, due to reduced external fat depth and marbling (Marshall, 1994).

The objective of this experiment was to compare the live and carcass production traits of progeny from three high-performing purebred sires (Angus, Charolais, Simmental) with the progeny of a crossbred bull (14% Zebu, 86% Angus; ALPHA) born through SCNT from a carcass that graded P1.

## MATERIALS AND METHODS

The cloning procedure that created ALPHA was completed under the West Texas A&M University Institutional Animal Care and Use Committee (IACUC) protocol 031114. Progeny from the current experiment were generated from semen provided to Cactus Feeders (owner of dams) to be used for artificial insemination. Live cattle in this experiment were under direct care and supervision of Cactus Feeders. All live animal experimental procedures followed the guidelines described in the Guide for the Care and Use of Agricultural Animals in Agricultural Research and Teaching (FASS, Savoy, IL).

### Sire Cloning Procedure

One sire used in this experiment, “ALPHA”, was a product of SCNT performed with a somatic tissue sample from a steer carcass that graded USDA prime and yield grade (YG) 1 at the commercial harvest facility in which the carcass was discovered in 2010. The carcass from which ALPHA was cloned was selected based on phenotypic attributes that are of value in the U.S. beef population, including: LM area of 102.6 cm^2^, subcutaneous fat thickness of 1.1 cm, 354.7 kg HCW, calculated YG of 1.98, and USDA marbling score of slightly abundant 70 (Slab^70^, USDA Prime). In addition, DNA from ALPHA was sent to Zoetis Inc. (Kalamazoo, MI) for genotypic analysis using the HD50K test, ranking ALPHA in the 6, 8, 10, and 30 percentiles for tenderness, palatability, feed efficiency, and marbling, respectively. Utilizing BreedSure genetic testing (ViaGen LLC; Cedar Park, TX), ALPHA’s breed percentage was 86% Angus and 14% Zebu, reporting ALPHA as an Ultrablack (Angus crossed with Brangus × Black Angus). ViaGen LLC, a commercial livestock and pet cloning company, performed the enucleation and SCNT transfer procedure. The embryo was placed into a recipient cow that carried out gestation at the West Texas A&M University Nance Ranch (Canyon, TX). ALPHA was born in July 2012, and first milk was administered through a calf bottle with no maternal contact. ALPHA was raised and cared for at the West Texas A&M University Nance Ranch.

### Dam Randomization and Breeding

British × Continental beef cows (*N* = 1269) representing a variety of breeds and crosses with a wide age range, sourced from multiple producers and auction markets, were utilized in this experiment. Within a 34-h time period, cows were artificially inseminated (AI) after timed AI synchronization, with semen collected from one of three selected purebred sires of the Charolais, Simmental, and Black Angus breeds, or from ALPHA. The three sires selected for use to compare to ALPHA in this terminal sire test were chosen based on their carcass merit. Cows entered the AI boxes at random; 10 straws of semen were thawed and inseminated per sire before cycling to the next sire in the four-sire rotation. A total of 312 straws of ALPHA semen, 300 straws of the Angus sire, 299 straws of the Simmental sire, and 358 straws of the Charolais sire semen were delivered to the dams by four trained AI technicians.

The full French Charolais sire (CHAR), registered in the American International Charolais Association as Anjou Pure Power 184Y FFM837595, ranked in the 4th percentile of his breed for ribeye area (REA) and in the 15th percentile for carcass weight. The Simmental sire (SIM), reported by ABS Global (2018) as Dikemans Sure Bet 29SM0390, ranked in the 4th percentile of his breed for marbling. The Angus sire (ANG), Rito Revenue 5M2 29AN1688, ranked in the top 1% of his breed for marbling. These sires were selected for use in this experiment because of their phenotypic carcass traits and the access to and availability of semen for each sire.

Cleanup bulls (Simmental × Angus) were turned out 4 d after AI to ensure females were bred. During gestation, dams were confined to traditional outdoor feedlot pens and limit fed a balanced daily ration to support gestation. There were 869 live calves born from the 1269 dams; of the live calves born, ALPHA, ANG, CHAR, and SIM sired 104, 134, 157, and 124 calves, respectively. The remaining 350 calves were excluded from analyses, including calves sired by the cleanup bulls (*N* = 314) and offspring with inconclusive parentage results (*N* = 36).

### Calf Identification and Weaning

Progeny from these matings (*N* = 876) were born in fall 2016 at a confined cow/calf operation located in Syracuse, KS. Calves were visually identified at parturition with birthdate; DNA samples of individual calves were collected at intermediate processing and matched with an EID tag and individual visual ear tag. Sire semen was sent to Quantum Genetix (Saskatoon, AB, CAN) for SNP parentage determination of each progeny.

At birth, feedyard personnel evaluated calves for overall health and dams on maternal ability. Calves born to unfit dams or in poor health condition (*N* = 69), were transported to a dairy calf ranch located in Syracuse, KS, where they received elevated care to improve upon health. Of the calves raised at the calf ranch, ALPHA and the SIM sired the fewest (2 calves per sire), the ANG sired 3 calves, the CHAR sired 11 calves, and the remaining 51 calves were sired by the cleanup bulls.

Calves in adequate health condition (*N* = 807) remained at Syracuse Feedyard and were weaned in early January 2017 (mean WW = 214 ± 5 kg) at approximately 4 mo of age. At time of weaning, calves were administered a modified live vaccine for prevention of infectious bovine rhinotracheitis, bovine viral diarrhea, parainfluenza-3, and bovine respiratory syncytial virus (Pyramid 5, Boehringer Ingelheim Vetmedica, Inc., St. Joseph, MO), a killed vaccine for clostridial toxoids and *Histophilus somni* (Ultrabac 7/Somubac, Zoetis Inc.), orally dosed with a bovine dewormer to target brown stomach worms (Synanthic, Boehringer Ingelheim Vetmedica, Inc.), administered an injectable solution for the treatment and control of gastrointestinal roundworms, lungworms, eyeworms, lice, and mites (Dectomax, Zoetis Inc.), and treated with a pour-on insecticide for control of flies (CyLence; Bayer HealthCare LLC, Shawnee Mission, KS). Postweaning, calves were fed a baseline growing ration, and the health of each individual animal was monitored daily by Syracuse Feedyard personnel.

A snowstorm struck Syracuse, KS, in May 2017 with a combination of wind and snowfall up to 30 inches in depth, causing fatality (*N* = 114; ALPHA = 8; ANG = 16; CHAR = 24; SIM = 11; cleanup bulls = 55) in weaned calves located at Syracuse Feedyard, thus removing these individuals from the remainder of the experiment. Calves housed at the calf ranch were evaluated for overall health by feedyard personnel, and if deemed healthy, were transferred to Syracuse Feedyard once they reached a minimum body weight of 159 kg, with weaning weight (WW) data absent for these calves.

### Processing and Sorting Calves

In mid-June 2017, calves (*N* = 754) were sorted by sex for shipping to Ulysses, KS, on the following day. Upon arrival at Ulysses Feedyard, the heifers (*N* = 370) and steers (*N* = 373) were sorted five-ways per sex (totaling 10 pens) based on a proprietary combined frame score and weight measurement sorting system (Garrison, 2005). The sorting system projected days on feed (DOF) by creating an algorithm based on sex of the animal, hip height, hip length, and weight at feedlot arrival processing to predict appropriate finish weight for each animal.

Steers and heifers were implanted based on arrival weight at entry into the feedlot. The lighter weight (277 ± 27.6 kg) pens of steers (three pens) were initially implanted with 80 mg trenbolone acetate and 16 mg estradiol (Revalor-IS; Merck Animal Health, Summit, NJ) and reimplanted approximately 70 d into the finishing period with 200 mg trenbolone acetate and 40 mg estradiol (Revalor-XS; Merck Animal Health). The heavier weight (323 ± 24.4 kg) pens of steers (two pens) were implanted with 200 mg trenbolone acetate and 40 mg estradiol (Revalor-XS; Merck Animal Health). The lighter weight (253 ± 25.3 kg) pens of heifers (three pens) were initially implanted with 80 mg trenbolone acetate and 8 mg estradiol (Revalor-IH; Merck Animal Health) and reimplanted approximately 80 d into the finishing period with 200 mg trenbolone acetate and 20 mg estradiol (Revalor-XH; Merck Animal Health). The heavier weight (298 ± 29.3 kg) pens of heifers (two pens) were implanted with 200 mg trenbolone acetate and 20 mg estradiol (Revalor-200; Merck Animal Health).

Heifers were rectally palpated with an ultrasound probe to verify absence of pregnancy. For heifers in which palpation indicated pregnancy (*N* = 3), a combination abortifacient (Lutalyse; Zoetis Inc.) and anti-inflammatory injectable (dexamethasone; Phoenix Pharmaceutical Inc., Burlingame, CA) were administered to stimulate abortion. Males identified as intact bulls (*N* = 11) were removed from the experiment.

Morbidity was documented throughout life for each individual animal, based on the feedyard doctor tag. Diet composition was equivalent across all weight and sex groups, with an industry standard growing ration followed by a finishing ration typical for the Great Plains region. DOF varied for the 10 pens of cattle, as the proprietary cattle sorting system was utilized to predict target finish weight based on hip height, hip length, and body weight at feedyard arrival.

### Harvest and Carcass Grading

Cattle were harvested within their five-way (steers and heifers separate) sort pens (10 total) during seven harvest dates at a commercial beef processor (USDA Est. #278) located 83 km from the experiment feedyard. At harvest, individual animal identification was recorded, and individual EID tags were scanned using a handheld Allflex RFID stick reader (Allflex ISO RFID Stick Reader, RS340-60, France). Carcass data on individual animals were tracked through the harvest floor by placing a shroud pin with a sequence number tag into the brisket of the carcass. Two trained employees of the Beef Carcass Research Center (Canyon, TX) recorded frequency and severity of liver abnormality (Brown and Lawrence, 2010) and evaluated lung health utilizing a lung scoring system reported by Tennant et al. (2014).

HCW (kg) was recorded from the beef processor carcass identification tag and referenced back to processor records. Carcasses were chilled 28 h postmortem and QG, 12th rib fat thickness, and longissimus muscle area (LMA) were assessed using the VBG2000 grading camera (VBG2000, E + V Technology, Oranienbury, Germany). YG was calculated utilizing HCW, LMA, and fat thickness observed from the camera data, with a constant value of 2.5% for kidney, pelvic, and heart fat estimation.

Total carcass value and carcass value per century weight (cwt) were calculated using a carcass base price of US$192.42/cwt derived from the U.S. Department of Agriculture Agricultural Marketing Service (AMS, 2018), and carcass value pricing (Table 1) representative of the 2017 calendar year.

**Table 1.** Carcass value grid based on average price for 2017 derived from the U.S. Department of Agriculture Agricultural Marketing Service

2017 Carcass base price	HCW, kg	Quality grade	Yield grade
	181–227 (−28.58)		1.0–1.9 (+3.69)
	227–250 (−20.54)		2.0–2.5 (+2.00)
	250–272 (−8.00)	Prime (+13.12)	2.6–3.0 (+1.62)
**192.42/cwt**	**273–408 (0.00)**	**Choice (0.00)**	**3.1–3.9 (0.00)**
	409–454 (−1.28)	Select (−11.44)	4.0–4.9 (−11.61)
	454–476 (−7.29)	Standard (−28.68)	5.0/up (−16.89)
	>476 (−23.70)		

### Warner-Bratzler Shear-Force Analysis

Using the PROC POWER function (α = 0.05; β = 0.80; σ= 1.2) in SAS (Version 9.3, 2011; SAS Institute Inc., Cary, NC), three commodity Choice carcasses per sire were selected, for a total of 12 carcasses per pen to follow through fabrication (48 h postmortem) and collect an IMPS 180 strip loin for Warner-Bratzler shear force (WBSF) analysis. Due to these selection criteria, the number of loins collected from each pen per sire were not equal.

Strip loins were aged 14 d prior to freezing and stored at the West Texas A&M University Meat Lab (Canyon, TX). Frozen strip loins were cut into 2.54 cm-thick steaks, with the second steak from the cranial end utilized for WBSF. Individual steaks remained frozen until October 2018, when they were thawed for WBSF analysis to obtain an objective measure of tenderness. One day postthaw, WBSF was completed according to the American Meat Science Association (AMSA, 2015) guidelines.

### Statistical Analysis

A completely randomized experimental design structure was used, with individual animal as the experimental unit. Interval data were analyzed using the MIXED procedure of SAS (SAS Institute, Inc., Cary, NC); the model included the fixed effect of sire, and the random effects of pen. Interval data included live cattle performance traits (WW, morbidity and mortality, DOF, days of age at harvest), carcass traits (HCW, backfat, LMA, YG, marbling, EBF), carcass value, and WBSF analysis. Frequency outcomes were analyzed using the PROC GLIMMIX procedure of SAS and were analyzed independently for sex. Frequency outcomes included abscessed liver, lung lesions, QG distribution, and YG distribution.

Multiple comparison means among sires were analyzed using the LSMEANS procedure of SAS, and means were separated when significant (*P ≤ *0.05) using the PDIFF option. Data were subjected to analyses of covariance, which included the constant endpoints of 200 DOF, HCW of 400 kg for steers and 375 kg for heifers, backfat thickness of 1.3 cm, and marbling score of Modest^00^. Significance was considered at α ≤ 0.05 and a tendency was considered at 0.05 < α ≤ 0.10.

## RESULTS AND DISCUSSION

A summary of the variation among sire breeds for heifer and steer DOF, days of age at harvest, carcass traits, and carcass value is reported in Table 2. This summary reports observed trait outcomes as an average, the standard deviation, and range that was observed for heifer and steer offspring of the four sires utilized in this terminal sire test.

**Table 2.** Summary of the variation among sire breeds for days on feed, carcass traits, and carcass value for heifers and steers from different sires in a terminal sire test

	Mean	Standard deviation	Minimum	Maximum
Heifers				
Days on feed^1^	194	16.9	175	228
Days of age at harvest	470	17.5	428	525
HCW, kg	374	30.0	260	446
Backfat, cm	1.44	0.464	0.48	2.77
LMA, cm^2^	95.46	9.222	67.68	121.87
Calculated yield grade^2^	2.82	0.785	0.800	4.98
Marbling score^3^	504	101.7	334	814
Empty body fat^4^, %	30.29	3.202	23.41	39.67
Carcass value, $	1685.10	143.336	1120.10	2072.16
Carcass value, $/cwt	204.46	5.459	192.98	229.48
Steers				
Days on feed	213	22.5	186	256
Days of age at harvest	486	20.8	433	541
HCW, kg	428	33.6	278	508
Backfat, cm	1.60	0.517	0.26	3.20
LMA, cm^2^	96.70	7.98	76.32	117.06
Calculated yield grade	3.36	0.803	5.50	1.38
Marbling score	498	102.0	310	837
Empty body fat, %	31.92	3.426	22.52	41.85
Carcass Value, $	1894.99	133.237	1246.75	2187.43
Carcass Value, $/cwt	199.70	11.247	153.98	226.98

^1^Days on feed: calculated from date of Ulysses, KS, feedlot arrival to date of harvest.

^2^USDA yield grade equation: 2.50 + (0.98425 × 12th rib fat, cm) + (0.2 × KPH, %) + 0.00837 × HCW, kg) – (0.0496 × LM area, cm^2^); USDA (2016).

^3^Marbling score: 400 = small^00^, minimum required for U.S. Low Choice; 500 = modest^00^, minimum required for U.S. Premium Choice.

^4^17.76207 + (4.68142 × 12^th^ rib fat, cm) + (0.01945 × HCW, kg) + (0.81855 × quality grade; 4 = Select, 5 = Choice -, 6 = Choice, 7 = Choice +, 8 = Prime) – (0.06754 × longissimus muscle area, cm^2^); Guiroy et al. (2002).

### Live Cattle Outcomes

Days in gestation did not differ (*P* = 0.44) between sire groups; weighted average gestation was 284 d. WW was lightest (*P* = 0.03) for ALPHA-sired calves (Table 3). No difference in WW was detected between progeny of the CHAR, ANG, or SIM sires. The WW outcomes for these three sires were not consistent with outcomes expected for their respective breeds, where previous literature reported Charolais-sired calves to have heavier WW than Angus-sired calves (Peacock et al., 1978; Nadarajah et al., 1984).

**Table 3.** Calf metrics by sire for live animal outcomes assessed from calf birth to shipping at Syracuse Feedyard, Syracuse, KS

Outcome	Alpha	Angus	Charolais	Simmental	SEM	*P-*value
*N*	104	134	157	124	–	–
Gestation, d	285	285	283	284	0.7	0.44
Male, %	51.9	48.5	45.9	55.6	–	–
Female, %	48.1	51.5	54.1	44.4	–	–
Weaning weight, kg	135.4^b^	141.8^a^	143.5^a^	145.8^a^	3.4	0.03
Morbidity, %	13.4	29.1	37.8	22.9	–	0.08
Mortality, %	10.4	13.6	17.3	8.6	–	0.33

^a,b^Means within a row lacking a common superscript letter differ (*P* ≤ 0.05).

Calf morbidity tended (*P *= 0.08) to be lower for offspring sired by ALPHA and higher for calves that were CHAR sired (Table 3). Based on the carcass composition of ALPHA, USDA P1, it may be hypothesized that cattle with this rare phenotype have not succumbed to viral or bacterial illness at any period in their lifetime (Gardner et al., 1999; Tennant et al., 2014).

DOF were calculated from date of Ulysses, KS, feedlot arrival to date of harvest. Based on the projections of the sorting system and the earlier-maturing physiology of British-type cattle, the ANG-sired heifers spent the least number of DOF (*P* < 0.01), not differing from SIM-sired heifers (Table 4). ANG-sired steers spent the least number of DOF (*P* < 0.01), not differing from SIM or CHAR-sired steers. ALPHA-sired steers spent the greatest number of DOF (*P* ≤ 0.01), which may have been influenced by the later-maturing nature of cattle with *Bos indicus* genetics (Hammack, 2007). When HCW was held constant at 400 kg, ANG-sired steers spent the least number of DOF, differing from all other sire groups (*P* = 0.01). When BF thickness was held constant at 1.3 cm, ALPHA-sired steers spent the greatest number of DOF, not differing from ANG-sired steers (*P* < 0.01). ALPHA-sired heifers spent the greatest number of DOF, not differing from CHAR-sired heifers, when BF endpoint was held constant (*P* < 0.01). The same pattern for heifers resulted when marbling was held constant, with ALPHA and CHAR-sired heifers not differing in number of DOF, and ANG-sired heifers spending the least number of DOF (*P* < 0.01). The response of covariate analysis consistently increased the number of DOF for both steers and heifers.

**Table 4.** Days on feed and days of age at harvest for heifers and steers from different sires utilized in a terminal sire test

		Days on feed^1^, d Covariate^2^ model				Days of age at harvest, d Covariate model			
	*N*	None	HCW	BF	Marb	None	HCW	BF	Marb
Heifers									
Alpha	41	200^a^	199^a^	201^a^	200^a^	473^a,b^	472^a,b^	474^a^	473^a,b^
Angus	58	185^b^	186^b^	188^c^	185^b^	463^c^	463^c^	466^b^	461^c^
Charolais	74	199^a^	198^a^	197^a,b^	199^a^	476^a^	475^a^	474^a^	477^a^
Simmental	50	191^b^	191^b^	192^b,c^	191^b^	467^b,c^	467^b,c^	468^a,b^	467^b,c^
SEM		2.2	2.1	2.4	2.3	2.3	2.2	2.5	2.4
*P*-value		<0.01	<0.01	<0.01	<0.01	<0.01	<0.01	0.05	<0.01
Steers									
Alpha	42	224^a^	227^a^	227^a^	224^a^	495^a^	497	497^a^	495^a^
Angus	50	206^b^	213^b^	218^a,b^	203^c^	483^b^	487	491^a,b^	479^c^
Charolais	50	210^b^	219^a,b^	207^b^	213^b,c^	486^b^	492	484^b^	490^a,b^
Simmental	59	213^b^	220^a,b^	218^b^	213^b^	483^b^	489	487^b^	484^b,c^
SEM		3.1	3.2	3.5	3.3	2.9	3.1	3.4	3.1
*P*-value		<0.01	0.01	<0.01	<0.01	0.02	0.11	0.02	<0.01

^1^Days on feed: calculated from date of Ulysses, KS, feedlot arrival to date of harvest.

^2^Covariate analysis included the constants of 200 days on feed (DOF), hot carcass weight (HCW) of 400 kg for steers and 375 kg for heifers, backfat (BF) thickness of 1.3 cm, and a marbling score (Marb) of modest^00^.

^a,b,c^Means within a column within a sex lacking a common superscript letter differ (*P* ≤ 0.05).

CHAR-sired heifers were the oldest chronological age at time of harvest, not differing from ALPHA-sired heifers (*P* < 0.01), and ALPHA-sired steers were the oldest chronological age at time of harvest (*P* = 0.02; Table 4). When HCW was held constant, days of age at harvest did not differ between sire groups for steers (*P* = 0.11) suggesting that steers would be of similar age when they reached a common HCW of 400 kg. The marbling covariate demonstrated that to achieve a marbling endpoint of modest^00^, ANG-sired steers and heifers could have been harvested earlier, and CHAR-sired steers and heifers required a later harvest date to achieve the modest^00^ marbling endpoint.

In the United States, the average age for a feedlot animal at harvest is typically 17 mo or 510 d (NASEM, 2016). Cattle in this experiment were harvested at 15.5 to 16 mo of age for heifers and steers, respectively. The sire genetics utilized in this experiment were superior in terms of performance and carcass merit, and the sorting system projected that cattle with these high-performing genetics would reach their target body composition at an earlier age.

### Carcass Outcomes

Overall, the observed carcass traits for progeny from all sires utilized in the experiment were reflective of the carcass outcomes expected for each sire’s respective breed. Keeping in mind that this experiment compares offspring from four different sires and is not a breed comparison test. HCW did not differ for heifers from different sires (*P* = 0.81), nor did HCW differ when DOF was held constant (*P* = 0.67; Table 5). However, when backfat was held constant, CHAR-sired heifers were heavier (*P* < 0.01) than heifers from the other three sires. When marbling was used as a covariate, there was a tendency (*P* = 0.09) for CHAR-sired heifers to have the heaviest HCW. At harvest, HCW differed for steers from different sire groups (*P *= 0.04), where CHAR-sired steers were the heaviest and ALPHA-sired steers were the lightest but not different from the ANG-sired steers or SIM-sired steers. When DOF was held constant, the same outcome occurred, where CHAR-sired steers were heaviest in HCW, and ALPHA-sired steers were the lightest (*P* = 0.04). Lighter HCW in ALPHA-sired steers may be explained by the HCW of the steer in which ALPHA was cloned (354.7 kg), where his *Bos indicus* genetics may have influenced a lighter HCW, supported by findings of Crouse et al. (1989) where *Bos indicus* crossbred groups were lighter in weight than *Bos taurus* crossbred groups.

**Table 5.** Carcass outcomes including hot carcass weight (HCW), backfat, and longissimus muscle area (LMA) for steers and heifers from different sires in a terminal sire test

		HCW, kg Covariate^1^ model				Backfat, cm Covariate model				LMA, cm^2^ Covariate model				
	*N*	None	DOF	BF	Marb	None	DOF	HCW	Marb	None	DOF	HCW	BF	Marb
Heifers														
Alpha	41	368	368	363^b^	368	1.48^b^	1.48^b^	1.51^b^	1.47^b^	95.2^b^	95.2^b^	96.1^b^	96.2^a,b^	95.4^b^
Angus	58	369	367	354^b^	362	1.83^a^	1.79^a^	1.82^a^	1.69^a^	90.1^c^	89.6^c^	90.9^c^	92.8^b^	92.0^b^
Charolais	74	373	373	380^a^	377	1.07^c^	1.07^c^	1.07^c^	1.15^c^	99.9^a^	99.8^a^	100.2^a^	98.6^a^	98.8^a^
Simmental	50	370	370	364^b^	370	1.53^b^	1.51^b^	1.53^b^	1.54^b^	93.2^b,c^	92.9^b^	93.9^b^	94.3^b^	93.2^b^
SEM		7.1	4.1	6.5	7.5	0.05	0.05	0.05	0.05	1.6	1.3	1.1	1.7	1.5
*P*-value		0.81	0.67	<0.01	0.09	<0.01	<0.01	<0.01	<0.01	<0.01	<0.01	<0.01	0.02	<0.01
Steers														
Alpha	42	414^b^	425^b^	406^b,c^	413^c^	1.53^b^	1.62^b^	1.44^b^	1.52^b^	96.8^b^	97.6^b^	95.4^b^	97.3^b^	96.9^b^
Angus	50	420^a,b^	431^a,b^	394^c^	414^b,c^	2.03^a^	2.12^a^	1.94^a^	1.88^a^	90.8^c^	91.4^c^	88.5^c^	92.4^c^	92.2^c^
Charolais	50	431^a^	442^a^	442^a^	436^a^	1.06^c^	1.15^c^	0.90^c^	1.19^c^	103.7^a^	103.2^a^	99.3^a^	102.2^a^	101.4^a^
Simmental	59	426^a,b^	438^a^	416^b^	427^a,b^	1.62^b^	1.71^b^	1.48^b^	1.63^b^	96.4^b^	97.1^b^	93.5^b^	97.0^b^	96.2^b^
SEM		7.6	5.1	5.9	8.1	0.08	0.07	0.05	0.08	1.1	1.0	0.9	1.3	1.2
*P*-value		0.04	0.04	<0.01	<0.01	<0.01	<0.01	<0.01	<0.01	<0.01	<0.01	<0.01	<0.01	<0.01

^1^Covariate analysis included the constants of 200 days on feed (DOF), hot carcass weight (HCW) of 400 kg for steers and 375 kg for heifers, backfat (BF) thickness of 1.3 cm, and a marbling score (Marb) of modest^00^.

^a,b,c^Means within a column within a sex lacking a common superscript letter differ (*P* ≤ 0.05).

When backfat was held constant, offspring from the CHAR sire were the heaviest (*P* < 0.01), and offspring from the ANG sire were the lightest but not differing from ALPHA-sired offspring. When marbling was held constant, there was a tendency (*P* = 0.09) for CHAR-sired heifers to have the heaviest HCW, and CHAR-sired steers to have the heaviest HCW (*P *< 0.01). The HCW outcomes for cattle in this experiment were consistent with Wheeler et al. (2005), who observed that British sire breeds, such as Angus, were lighter in HCW than that of Continental sire breeds, such as Charolais. Similarly, Morris et al. (1987) observed that heavier breeds of cattle, such as Charolais, are generally leaner and larger in frame, resulting in reduced backfat thickness and thus additional HCW is required to achieve greater rates of marbling.

Backfat differed for heifers from different sires (*P* < 0.01) with ANG-sired heifers having the greatest backfat depth and CHAR-sired heifers having the least (Table 5). ALPHA- and SIM-sired heifers did not differ in backfat thickness and were intermediate to heifers sired by ANG and CHAR sires. The same outcome was observed for steers (*P *< 0.01), with ANG-sired steers having the greatest backfat depth and CHAR-sired steers having the least. Although the DOF, HCW, and marbling covariates slightly altered BF thickness, the same patterns were observed between sire groups, where ANG-sired offspring were consistently the fattest at time of harvest, and offspring from the CHAR sire were consistently the leanest at time of harvest.

LMA (cm^2^) differed for heifers (*P* ≤ 0.02) and steers (*P* < 0.01) from different sire groups, with CHAR-sired calves measuring the largest LMA, and ANG-sired calves measuring the smallest LMA (Table 5). ALPHA- and SIM-sired calves did not differ in LMA. The LMA outcomes observed in this experiment are consistent with that of Wheeler et al. (2005) who reported that, when compared to British sire breeds, Continental sire breeds had larger LMA. Although the DOF, HCW, BF and marbling covariates slightly altered mean LMA, the same patterns were observed between sire groups, where CHAR-sired offspring observed the largest LMA, and ANG-sired offspring observed the smallest LMA at time of harvest.

USDA YG and marbling scores (Table 6) of the sire groups followed trends that have been observed in previous literature for each sire’s respective breed (Marshall, 1994; Sexten et al., 2012). ANG-sired steers (*P *< 0.01) and heifers (*P* < 0.01) had the highest USDA calculated YG, therefore, lowest cutability, and greatest marbling scores, which was previously reported by Sexten et al. (2012). CHAR-sired steers and heifers had the lowest USDA calculated YG, therefore greatest cutability, and lowest marbling scores, similar to results reported by Marshall (1994) and Sexten et al. (2012). ALPHA-sired calves were similar to SIM-sired calves in calculated YG and marbling score. When backfat was held constant, calculated YG did not differ between sire groups for heifers (*P* = 0.45) or steers (*P* = 0.14), indicating that backfat thickness has the greatest impact on calculated YG outcomes. When marbling was held constant, calculated YG reduced for ANG-sired offspring and increased for CHAR-sired offspring (*P *< 0.01), which was expected, based on the antagonistic relationship between backfat and marbling, previously described by Cundiff (1992).

**Table 6.** Carcass outcomes including USDA calculated yield grade, marbling score, and empty body fat percentage for heifers and steers from different sires in a terminal sire test

		Calculated yield grade^1^ covariate^2^ model					Marbling score^3^ covariate model				Empty body fat^4^, %, covariate model				
	*N*	None	DOF	HCW	BF	Marb	None	DOF	HCW	BF	None	DOF	HCW	BF	Marb
Heifers															
Alpha	41	2.82^b^	2.82^b^	2.86^b^	2.55	2.79^b^	509^b^	509^b^	513^b^	493^b^	30.4^b^	30.4^b^	30.7^b^	29.3^b^	30.3^b^
Angus	58	3.43^a^	3.39^a^	3.43^a^	2.64	3.16^a^	586^a^	589^a^	588^a^	540^a^	33.1^a^	32.9^a^	33.1^a^	29.7^a^	31.4^a^
Charolais	74	2.22^c^	2.22^c^	2.23^c^	2.57	2.39^c^	446^c^	447^c^	448^c^	468^b^	27.8^c^	27.7^c^	27.8^c^	29.2^b^	28.7^c^
Simmental	50	2.99^b^	2.97^b^	2.99^b^	2.64	3.02^a,b^	492^b^	494^b^	495^b^	473^b^	30.7^b^	30.6^b^	30.7^b^	29.2^b^	30.8^a,b^
SEM		0.09	0.09	0.08	0.05	0.08	12.3	13.0	13.6	13.2	0.34	0.35	0.30	0.15	0.30
*P*-value		<0.01	<0.01	<0.01	0.45	<0.01	< 0.01	< 0.01	< 0.01	< 0.01	< 0.01	< 0.01	< 0.01	0.03	< 0.01
Steers															
Alpha	42	3.16^b^	3.31^b^	3.03^b^	2.85	3.14^c^	504^b^	495^b^	498^b^	485^b^	31.4^b^	31.9^b^	30.7^b^	30.0	31.3^c^
Angus	50	4.05^a^	4.16^a^	3.87^a^	3.01	3.76^a^	587^a^	582^a^	578^a^	525^a^	35.0^a^	35.5^a^	34.2^a^	30.4	33.3^a^
Charolais	50	2.59^c^	2.71^c^	2.31^c^	2.92	2.81^d^	420^c^	413^c^	407^c^	440^c^	28.5^c^	29.0^c^	27.2^c^	29.9	29.9^d^
Simmental	59	3.40^b^	3.53^b^	3.16^b^	2.96	3.42^b^	489^b^	481^b^	478^b^	463^b,c^	32.0^b^	32.5^b^	30.9^b^	30.0	32.1^b^
SEM		0.11	0.10	0.08	0.05	0.13	14.2	14.9	15.9	16.29	0.46	0.43	0.32	0.14	0.52
*P*value		<0.01	<0.01	<0.01	0.14	<0.01	<0.01	<0.01	<0.01	<0.01	<0.01	<0.01	<0.01	0.08	<0.01

^1^USDA yield grade equation: 2.50 + (0.98425 × 12th rib fat, cm) + (0.2 × KPH, %) + 0.00837 × HCW, kg) – (0.0496 × LM area, cm^2^); USDA (2016).

^2^Covariate analysis included the constants of 200 days on feed (DOF), hot carcass weight (HCW) of 400 kg for steers and 375 kg for heifers, backfat (BF) thickness of 1.3 cm, and a marbling score (Marb) of modest^00^.

^3^Marbling score: 400 = small^00^, minimum required for U.S. Low Choice; 500 = modest^00^, minimum required for U.S. Premium Choice.

^4^17.76207 + (4.68142 × 12th rib fat, cm) + (0.01945 × HCW, kg) + (0.81855 × quality grade; 4 = Select, 5 = Choice −, 6 = Choice, 7 = Choice +, 8 = Prime) – (0.06754 × longissimus muscle area, cm^2^); Guiroy et al. (2002).

^a,b,c,d^Means within a column within a sex lacking a common superscript letter differ (*P* ≤ 0.05).

Empty body fat (EBF) percentage was computed from carcass metrics collected during harvest according to the EBF equation provided by Guiroy et al. (2002). Percentage EBF differed between sire groups for heifers and steers (*P* < 0.01); ANG-sired heifers and steers exhibited the highest EBF percentage and CHAR-sired heifers and steers exhibited the least EBF percentage, with ALPHA- and SIM-sired calves exhibiting similar EBF percentage (Table 6). When backfat was held constant for steers, there was a tendency (*P* = 0.08) for EBF to differ between sire groups, suggesting that backfat depth has a sizeable effect on EBF percentage. The outcomes for both heifers and steers agreed with Guiroy et al. (2001), who reported that percentage EBF increased with increased marbling, where Angus-sired calves had the greatest EBF and Charolais-sired calves had the least EBF percentage.

ANG-sired calves had the highest frequency of USDA Prime, not differing from the other sire groups (*P ≤ *0.25), and subsequently, the highest frequency of USDA YG 4’s, with ANG-sired steers differing from all other sire groups (*P* < 0.01; Table 7). CHAR-sired calves had the greatest carcass cutability as represented by the greatest frequency of YG 1 and 2 carcasses, differing in the frequency of USDA YG 1’s from all other sire groups (*P* < 0.01). ALPHA-sired offspring combined marbling with cutability, with ALPHA-sired heifers and steers having the greatest frequency of USDA QG Choice and YG 2 and 3 carcass outcomes.

**Table 7.** Frequency of USDA carcass quality and yield grade outcomes of heifers and steers from different sires in a terminal sire test

		Quality grade, %				Yield grade, %				
	*N*	Prime	CAB^1^	Choice	Select	1	2	3	4	5
Heifers										
Alpha	41	2.4	42.9^a^	47.6^b^	7.1	2.4^b^	71.4^a^	26.2^a,b^	0	0
Angus	58	19.0	43.1^a^	31.0^b^	6.9	1.7^b^	31.0^c^	46.6^a^	20.7	0
Charolais	74	0	1.4^b^	79.7^a^	18.9	47.3^a^	47.3^b,c^	5.4^c^	0	0
Simmental	50	0	48.1^a^	50.0^b^	1.9	7.7^b^	57.7^a,b^	23.1^b^	0	0
*P*-value		0.25	<0.01	<0.01	0.06	<0.01	<0.01	<0.01	0.66	1.0
Steers										
Alpha	42	2.4	35.7	59.5^a^	2.4^b^	2.4	35.7^a,b^	57.1^a^	4.8^b^	0
Angus	50	22.5	42.9	32.7^b^	2.0^b^	0	2.0^c^	44.9^a^	46.9^a^	6.1
Charolais	50	0	0	70.0^a^	28.0^a^	20.0	56.0^a^	22.0^b^	0^b^	0
Simmental	59	0	35.1	54.4^a^	10.5^b^	3.5	29.8^b^	54.4^a^	12.3^b^	0
*P*-value		0.19	0.85	0.02	0.01	0.06	<0.01	0.02	<0.01	1.00

^1^CAB: Certified Angus Beef; brand in which subprimals and retail cuts are marketed; carcasses meeting 10 quality standards as outlined and determined by the brand.

^a,b,c,d^Means within a column within a sex lacking a common superscript letter differ (*P* ≤ 0.05).

Liver (*P* ≥ 0.56) and lung health (*P* ≥ 0.27) outcomes did not differ between sire groups for steers or heifers (Table 8). Livers were reported as a frequency of all liver outcomes that had an active liver abscess, with CHAR-sired heifers (*P* = 0.83) and ALPHA-sired steers (*P* = 0.56) having the highest numerical percentage of liver abscess, and ALPHA-sired heifers and CHAR-sired steers having the lowest numerical percentage of liver abscess. These results suggest that breed type of the sires utilized in this experiment did not influence abscess outcome frequency. The frequency of liver abscess outcomes across the entire experiment population ranged from 4.8% to 16.7% (average 9.1%); these numbers are consistent with Herrick et al. (2022) who reported total liver abscess rate at 15.5% for fed-beef in the High Plains region. Lung scores were reported as a frequency of all lung outcomes that were nonnormal. Of the lungs evaluated in this experiment, a low frequency of cattle scored a three (consolidation of lung lobes involving greater than 50% of lung tissue). Tennant et al., (2014) reported that cattle with greater than 50% consolidation of lung tissue had significantly reduced HCW when compared to other lung health scores.

**Table 8.** Abscessed liver frequency and lung health for heifers and steers from different sires in a terminal sire test

		Abscessed liver, %	Lung score^1^, %				
	*N*		1	2	3	M	E
Heifers							
Alpha	41	4.8	5.3	7.9	0	23.7	13.2
Angus	58	6.9	6.9	8.6	0	34.5	20.7
Charolais	74	9.5	5.4	5.4	2.7	21.6	13.5
Simmental	50	7.7	11.5	5.8	1.9	25.0	17.3
*P*-value		0.83	0.59	0.88	0.99	0.42	0.68
Steers							
Alpha	42	16.7	11.9	7.1	0	16.7	16.7
Angus	50	10.2	4.1	10.2	0	32.7	6.1
Charolais	50	8.0	2.0	4.0	0	18.0	14.0
Simmental	59	8.8	8.8	0	0	21.1	15.8
*P*-value		0.56	0.31	0.72	1.00	0.27	0.45

^1^Lung health: 1 = consolidation of lung lobes involving 5% to 15% of lung tissue; 2 = consolidation of lung lobes involving 15% to 50% of lung tissue; 3 = consolidation of lung lobes involving greater than 50% of lung tissue; M = minor fibrin tag formation on lung tissue; E = extensive fibrin tag formation on lung tissue; Tennant et al. (2014).

Based on the results from this experiment, ALPHA-sired calves were most similar in carcass outcomes to SIM-sired calves. ALPHA-sired calves were not on the extreme ends of YG, QG, LMA, or fat thickness, suggesting that calves sired by the P1 carcass clone are intermediate in frame and carcass outcomes. The economic benefit of these intermediate carcass outcomes is reflected in the total carcass value of ALPHA-sired calves. Numerically, ANG-sired heifers (*P* = 0.87) and SIM-sired steers (*P* = 0.73) were worth the most in total carcass value (Table 9). When HCW was held constant, heifers sired by ANG and ALPHA were worth the greatest total carcass value (*P *= 0.05). When backfat was held constant, CHAR-sired heifers were worth the greatest total carcass value (*P* = 0.04), differing from all other sires. When marbling was held constant, CHAR-sired heifers were worth the greatest total carcass value, not differing from SIM-sired heifers (*P* = 0.02), and ANG-sired heifers were numerically worth the least. Steer total carcass value differed (*P* = 0.05) between sire groups when marbling was held constant, where CHAR-sired steers were worth the greatest dollar value, not differing from SIM- and ALPHA-sired steers, and ANG-sired steers were worth the least. These outcomes suggest that the premium attained for QG greatly impacts the total carcass value of feeder cattle in the United States.

**Table 9.** Total carcass value and carcass value per century weight (cwt) of heifers and steers from different sires utilized in a terminal sire test

		Total carcass value, $ covariate^1^ model						Carcass value, $/cwt covariate model			
	*N*	None	DOF	HCW	BF	Marb	None	DOF	HCW	BF	Marb
Heifers											
Alpha	41	1659	1657	1693^a,b^	1638^b^	1653^b^	204.6	204.6^a,b^	204.7	205.0^a,b^	204.2^a,b^
Angus	58	1682	1673	1704^a^	1624^b^	1622^b^	206.0	206.4^a^	206.0	207.2^a^	202.8^b^
Charolais	74	1676	1677	1683^b^	1703^a^	1707^a^	203.6	203.6^b^	203.6	203.0^b^	205.^5a^
Simmental	50	1669	1665	1684^b^	1642^b^	1669^a,b^	203.8	204.1^b^	203.8	204.4^b^	204.1^a,b^
SEM		29.9	19.8	6.2	28.2	32.9	0.7	0.8	0.7	0.8	0.6
*P*-value		0.87	0.90	0.05	0.04	0.02	0.06	0.04	0.06	<0.01	0.04
Steers											
Alpha	42	1887	1902	1829	1875	1878^a,b^	203.3	202.0	206.6	206.4^a^	202.7
Angus	50	1884	1893	1806	1853	1843^b^	200.5	199.5	204.5	209.4^a^	197.7
Charolais	50	1885	1895	1783	1895	1924^a^	196.6	195.5	202.0	194.1^c^	199.2
Simmental	59	1911	1924	1819	1896	1917^a^	200.9	199.4	205.5	204.9^b^	201.2
SEM		23.4	23.7	19.3	25.9	24.5	2.3	2.1	1.9	2.2	2.2
*P*-value		0.73	0.66	0.27	0.54	0.05	0.07	0.09	0.24	<0.01	0.21

^1^Covariate analysis included the constants of 200 days on feed (DOF), hot carcass weight (HCW) of 400 kg for steers and 375 kg for heifers, backfat (BF) thickness of 1.3 cm, and a marbling score (marb) of modest^00^.

^a,b,c^Means within a row lacking a common superscript letter differ (*P* ≤ 0.05).

Carcass value per cwt tended to be the greatest for ANG-sired heifers (*P* < 0.06; Table 9). When marbling was held constant, CHAR-sired heifers were worth the greatest value per cwt (*P* = 0.04), not differing from ALPHA- or SIM-sired heifers. ALPHA-sired steers tended (*P* = 0.07) to have the greatest carcass value per cwt compared to steers from other sires. When backfat was held constant, carcass value per cwt differed between steers from the four sire groups (*P* < 0.01), with ANG- and ALPHA-sired steers worth the greatest dollars per cwt. Calves sired by the ANG and CHAR were most greatly impacted by the carcass dollar value per cwt when backfat and marbling were held constant, this being reflective of the genetic tendency of Charolais to be a leaner breed, and Angus to be an earlier-maturing, high marbling breed.

There were no differences (*P* ≥ 0.14) in longissimus muscle tenderness based on the mean shear force (kg) value from the WBSF analysis (Table 10). Tenderness was evaluated in this experiment because it is well-defined in literature that cattle with *Bos indicus* genetics have reduced tenderness, indicated by increased WBSF values (Thrift and Thrift, 2002). Therefore, the Brahman breed association implemented a tenderness EPD to ensure that the Brahman breed was meeting tenderness specifications, and so that Brahman breeders could make breeding decisions based on expected tenderness outcomes. According to the tenderness standards developed by ASTM (2011), red meat that requires a shear force of less than 4.4 kg meets “Certified Tender” requirements, and red meat that requires a shear force of less than 3.9 kg achieves the “Certified Very Tender” moniker. Least squares means reported from progeny of each sire utilized in this experiment were less than 3.6 kg, classifying the majority of both heifers and steers from all sires as “Certified Very Tender”. Therefore, the *Bos indicus* genetics of ALPHA did not negatively impact meat tenderness in his progeny.

**Table 10.** Warner-Bratzler Shear Force outcomes of loin samples from heifer and steer carcasses for comparison in a terminal sire test

	*N*	Mean shear, kg	Tender, %	Very tender, %
Heifers				
Alpha	15	3.24	86.7	86.7
Angus	9	3.56	66.7	66.7
Charolais	15	2.97	100	93.3
Simmental	14	3.18	100	100
SEM		0.2	–	–
*P*-value		0.14	–	–
Steers				
Alpha	15	3.07	100	100
Angus	9	3.21	100	100
Charolais	15	3.27	93.3	86.7
Simmental	12	2.85	100	100
SEM		0.2	–	–
*P*-value		0.20	–	–

Findings from this experiment suggest that ALPHA-sired offspring have desirable and comparable carcass traits when compared to offspring of high-performing reference sires, included in the experiment because of their popularity and success as terminal sires within their respective breeds. The results from this experiment highlight the opportunity in selecting phenotypic carcass traits, and how selection for these traits can increase economic value of terminal offspring. We were able to create a sire from a cloned P1 carcass that produced offspring comparable in carcass outcomes and value to offspring of sires that have undergone multiple years of breeding selection. The results of this experiment suggest that there is economic benefit in selecting sires based on their known carcass outcomes, in this case, a clone from a P1 steer carcass, to maintain and improve on meat quality, while reducing overall waste fat from the carcass.
